# A182 EXPLORING THE LINK BETWEEN SPECIFIC MICROBIAL STRAINS AND ANXIETY AND DEPRESSION IN PATIENTS WITH ULCERATIVE COLITIS

**DOI:** 10.1093/jcag/gwad061.182

**Published:** 2024-02-14

**Authors:** B A Chiew, N Haskey, A Lewis, H Nadeem, S L Gold, L M Taylor, K McCoy, C Ohland, K McGregor, M Raman

**Affiliations:** University of Calgary Cumming School of Medicine, Calgary, AB, Canada; The University of British Columbia Okanagan Irving K Barber School of Arts and Sciences, Kelowna, BC, Canada; The University of British Columbia Okanagan Irving K Barber School of Arts and Sciences, Kelowna, BC, Canada; University of Calgary Cumming School of Medicine, Calgary, AB, Canada; Icahn School of Medicine at Mount Sinai, New York, NY; University of Calgary Cumming School of Medicine, Calgary, AB, Canada; University of Calgary Cumming School of Medicine, Calgary, AB, Canada; University of Calgary Cumming School of Medicine, Calgary, AB, Canada; York University, Toronto, ON, Canada; University of Calgary Cumming School of Medicine, Calgary, AB, Canada

## Abstract

**Background:**

Anxiety and/or depression (A/D) have been identified as significant co-morbidities of ulcerative colitis (UC), and even considered as extraintestinal manifestations, with emerging evidence to support the role for gut microbial dysbiosis in the natural history for both UC and A/D. However, there is a paucity of knowledge regarding the specific attributes of the gut bacteriome in individuals with UC with concomitant A/D.

**Aims:**

To explore the connection between the gut bacteriome and A/D in a cohort of patients with UC.

**Methods:**

This cross-sectional study included 29 participants diagnosed with UC either in remission or with mild to severe disease. To test for depression and anxiety, participants completed the Patient Health Questionnaire-8 (PHQ-8) and General Anxiety Disorder-7 (GAD-7) respectively. Participants provided a stool sample for microbiome analysis and the fecal bacteriome was assessed using metagenomic shotgun sequencing.

**Results:**

Among the 29 patients, 55% were female (n=16) and 45% were male (n=13), 34.5 % (n=10) had a Partial Mayo Score over 5 indicating moderate-severe disease activity (median 3, IQR 1-6). Mean age was 38.3 years (± 12.2 years). Sixty-nine percent were on 5-ASA, 42.9% on steroids, 31.0% on Anti-TNF, and 20.7% on immunomodulators. Mean FCP was 976.4 μg/g (± 1589.2). Forty-four percent (n=12) screened positive for anxiety with a median GAD-7 score of 5.0 (IQR: 1-8), while 26% screened positive for depression with a median PHQ-8 of 3 (IQR: 3-10). Principal Coordinate Analysis (PCoA) reveals distinct clustering between UC patients with or without A/D (Figure 1). Regarding the microbial analyses, the log fold change of *Firmicutes bacterium* CAG:424, *Holdemanella biformis*, *Blautia hansenii*, *Bifidobacterium pullorum*, *Anaerostipes caccae*, and *Ruminococcus gnavus* exhibited positive associations with both A/D, whereas *Roseburia sp.* CAG:303, *Prevotella copri*, and *Bacteroides stercoris* demonstrated negative associations. Uniquely, *Faecalicoccus pleomorphus* and *Lachnospira pectinoschiza* showed negative associations only with the PHQ-8.

**Conclusions:**

Our research affirms the presence of anxiety and depression in a cohort of UC patients. The severity of A/D measured by the GAD-7 and PHQ-8 were linked to the levels of specific fecal microbes. We introduce several novel species that warrant further examination in UC patients with and without A/D. As this was an exploratory study, the findings need replication in a larger sample size.

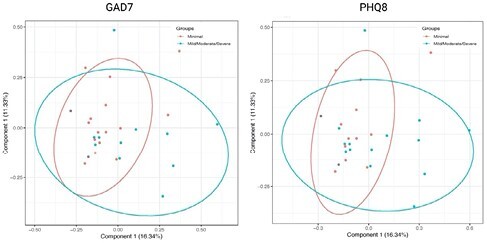

PCoA results reveal distinct bacterial clustering patterns between individuals with minimal anxiety/depression (absence) versus those with mild/moderate/severe anxiety and depression as measured by GAD-7 and PHQ-8.

**Funding Agencies:**

None

